# Levels of Essential and Trace Elements in Mozzarella Available on the Slovak Market with the Estimation of Consumer Exposure

**DOI:** 10.1007/s12011-023-03813-x

**Published:** 2023-08-18

**Authors:** Marcela Capcarova, Marcella Frigenti, Julius Arvay, Ivona Janco, Lubos Harangozo, Anna Bandlerova, Martina Sartoni, Alessandra Guidi, Robert Stawarz, Gregorz Formicki, Maria-Jose Argente, Peter Massanyi

**Affiliations:** 1 Institute of Applied Biology, Faculty of Biotechnology and Food Sciences, Slovak University of Agriculture in Nitra, Tr. A. Hlinku 2, 949 76 Nitra, Slovak Republic; 2 Department of Veterinary Sciences, University of Pisa, Viale Delle Piagge 2, 56124 Pisa, Italy; 3 Institute of Food Sciences, Faculty of Biotechnology and Food Sciences, Slovak University of Agriculture in Nitra, Tr. A. Hlinku 2, 949 76 Nitra, Slovak Republic; 4 Institute of Law, Faculty of European Studies and Regional Development, Slovak University of Agriculture in Nitra, Tr. A. Hlinku 2, 949 76 Nitra, Slovak Republic; 5 Institute of Biology, Pedagogical University of Cracow, Ul. Podchorazych 2, 30-084 Cracow, Poland; 6 Department of Agro-Food Technology, Universidad Miguel Hernández de Elche, 03312 Orihuela, Spain

**Keywords:** Mozzarella, Elements, Consumer exposure, PTWI

## Abstract

The aim of this study was to determinate the content of some elements in a specific dairy product, mozzarella, in a particular area of western Slovakia and to evaluate the estimation of the risk to the consumers based on the contribution to the provisional tolerable weekly intake. The consumption of mozzarella can contribute to the intake of important elements in the diet, such as calcium and magnesium, along with others. The contents of some toxic and trace elements were low and have not exceeded the permitted limit. In addition, the contribution to PTWI was found to be very low, which means that the consumption of mozzarella possesses no risk to humans. It is concluded that the data obtained in this study can help as a valuable addition to methodological and scientific material in the field of food safety of dairy products and their positive impact on human health.

## Introduction

Milk and its derivatives are essential constituents of human nutrition [1]. Among them, cheese is a highly nutritious and delicate food that contains almost all of the protein, essential minerals, fat, vitamins, and other important nutrients [2, 3]

To ensure its quality and safety, cheese producers must evaluate the relevant properties and health-related characteristics of the cheese [3]. At the same time, since cheese is an integral component of various food products, it is imperative for producers to manufacture cheese with technological and qualitative characteristics that cater to the intended end-use [4]. Several studies have demonstrated that the consumption of dairy products can contribute to reduce the risk of metabolic syndrome by influencing weight gain, lipid levels, blood pressure, and insulin sensitivity [5, 6], and the study by Samara et al. [7] found a positive correlation between dairy consumption and body mass index (BMI), weight, and triglyceride contents in women.

In human body, mineral elements account for 4% of total body mass and have structural, nutritional, biochemical functions that are exceedingly important for human health, mental, and physical. They serve as catalysts for numerous biological reactions, e.g., transmission of nerve impulses, muscle contraction, and utilization of nutrients food [8]. Essential elements in human nutrition are potassium (K), sodium (Na), chloride (Cl), calcium (Ca), selenium (Se), manganese (Mn), iodine (J), chromium (Cr), cobalt (Co), molybdenum (Mo), fluorine (F), nickel (Ni), silicon (Si), iron (Fe), copper (Cu), zinc (Zn), and boron (B) [9]. They are divided into two groups: major elements (K, Na, Cl, Ca, Mg, and P) that are in human body in concentration exceeds 0.01% of total body mass and trace elements (other 14 elements) that are present in much lower concentration [10]. Non-essential or toxic elements are lead (Pb), arsenic (As), and cadmium (Cd). Their presence even in low concentration is unwanted and can result in metabolic disorders [11]. Pb residues in milk products are of specific concern since they are largely consumed by children. It is considered as potential carcinogen and is related to the etiology of a number of diseases in the kidneys, cardiovascular system, nervous system, skeletal system, and blood [12, 13]. The content of trace elements in dairy products is influenced by the nutritional status of the animal, the stage of lactation, genetic factors, environment, manufacturing processes, and potential contamination during processing [9].

In our previous studies, we reported the presence of essential and trace elements as well as heavy metals in milk [14], dairy products as yogurt [15], and cottage [16] in the Slovak market with a consumer risk assessment. This study was conducted to measure and quantify the content of essential and toxic elements in mozzarella available on the Slovak market. Mozzarella is a highly popular cheese among consumers and belongs to the pasta filata family, which involves stretching the curd in hot water to obtain a smooth texture in cheese and the proper color. It is delicate, soft, unripened, and white and can be consumed shortly after manufacture [4, 17] being also the preferred cheese for use on pizza [3]. In addition, although mozzarella is becoming more popular as part of healthy food, there is only little data concerning this product.

The study focused on the analysis resulting from monitoring the consumption of a specific dairy product, mozzarella, in a particular area of western Slovakia, and the evaluation of the consumer risk estimation was carried out on the basis of the contribution to the provisional tolerable weekly intake.

## Material and Methods

The samples of commercially available mozzarella cheeses (*n* = 27) were purchased from different markets located in Nitra city, western Slovakia, in 2021. Sampled brands of mozzarella cheese were representative of the most consumed in the Slovak Republic. The cheeses were sold under well-known brand names. All samples came from imported products from abroad (Italy).

### Sampling

The samples were left in their original packages and transferred to the lab in an ice box. The material was carefully handled to avoid contaminating the products.

The samples were divided to 6 groups as follows: mozzarella classic (*n* = 9), mozzarella light (*n* = 9), mozzarella basil (*n* = 9), and 3 groups of whey (classic, light, and basil) from mozzarella (*n* = 27).

To prevent the risk of contamination, the use of glassware was reduced to a minimum. Sample of cheese was placed in a centrifugal tube and stored in a freezer (− 20 °C) until analysis.

### Laboratory Analysis

All the analyses of particular elements as aluminum (Al), arsenic (As), barium (Ba), calcium (Ca), chromium (Cr), copper (Cu), iron (Fe), potassium (K), magnesium (Mg), manganese (Mn), sodium (Na), nickel (Ni), lead (Pb), antimony (Sb), selenium (Se), strontium (Sr), and zinc (Zn) were carried out in the laboratory of Department of Chemistry of Faculty of Biotechnology and Food Sciences, and Agrobiotech, Slovak University of Agriculture in Nitra, Slovak Republic (Table 1).

**Table 1:** Parameters of the analytical procedures. Limits calculated for the analytical solution. Recovery and RSD calculated for quality check solutions of known concentrations

Element	Wavelength (nm)	LoD (µg/L)	LoQ (µg/L)	Recovery (%)	RSD (%)
Al	167.019	0.23	0.76	98.1	3.1
As	188.980	1.50	4.26	96.3	5.8
Ba	455.403	0.03	0.10	101.1	4.3
Ca	315.887	0.01	0.03	108.2	6.6
Cr	267.716	0.15	0.42	92.2	2.4
Cu	324.754	0.30	1.01	99.6	2.7
Fe	234.350	0.10	0.29	101.6	6.2
K	766.491	0.39	1.45	92.2	1.7
Mg	383.829	0.01	0.04	90.3	5.5
Mn	257.610	0.03	1.02	102.4	4.8
Mo	284.598	0.51	1.62	99.8	6.2
Na	589.592	0.15	0.46	94.1	6.9
Ni	231.604	0.30	0.99	104.9	4.1
Pb	220.353	0.82	2.01	93.7	6.2
Sb	206.834	2.00	5.62	100.3	6.3
Se	196.026	2.00	6.01	103.9	4.1
Sr	407.771	0.01	0.03	98.1	3.9
Zn	206.200	0.21	0.66	106.2	5.3

*LoD*, limit of detection; *LoQ*, limit of quantification

#### Analysis of the Content of Elements in Cottage Cheese by ICP-OES

The determination of individual elements in mozzarella samples was measured according to the method reported in the literature by Shah et al. [18] with minor modifications. Approximately 0.15–0.20 g of mozzarella samples was weighed using analytical balances ABT-120/5DM (Kern & Sohn, Germany) (accurate to 4 decimal places) and transferred to Teflon mineralization tubes. Mineralization was realized by a pressure microwave digestion on EthosOne (Milestone, Italy) in 5 mL concentrated nitric acid (69%) (Sigma Aldrich, Germany; trace purity) and 1 mL of 30% hydrogen peroxide (Sigma Aldrich, Germany; trace purity) with the addition of 2 mL of ddH2O (18.2 MΩ cm^−1^; 25 °C, Synergy UV, Merck Millipore, France). Following the decomposition, the digestate was filtered through a quantitative filter paper Filtrak 390 (Munktell & Filtrak, GmbH, Bärenstein, Germany) and filled with deionized water to 50 mL. Particular analysis was carried out on Agilent 720 ICP-OES spectrometer (Agilent Technologies Inc., Santa Clara, CA, USA) with axial plasma configuration and with auto-sampler SPS-3 (Agilent Technologies, Switzerland). Special experimental conditions were set as follows: RF power 1.45 kW, plasma gas flow 16.0 L/min, auxiliary gas flow 1.50 L/min, nebulizer gas flow 0.85 L/min, and CCD detector temperature − 35 °C. Signal acquisition time is 3 × 3 s for 3 replicates. All samples were analyzed for the concentration of 17 elements. ERM ®-CE278k (Mussel tissue; IRMM, Belgium) was used to check the measurement quality.

### Risk Assessment

The results were compared in regard to the safety thresholds set by law and risk assessments were evaluated based on EDI and PTWI.

The EDI (expected dietary exposure) for the investigated elements in mozzarella cheese (Ba, Ni, Sb, Sr, Ni, Pb, and Zn) was estimated per consumer (70 kg of body weight) according to the formula [19]:

$$\mathrm{EDI\mu g}/\mathrm{kgbw}/\mathrm{day}=\frac{{M}_{Me}\mathrm{ x }0.16}{70}$$

*M*_*Me*_ is mean concentration of metals in micrograms per milliliter.

0.16 kg of mozzarella cheese per day/per capita: according to the average consumption of milk in the Slovak Republic given by the Ministry of Agriculture and Rural Development of the Slovak Republic is 0.46 kg of milk and milk products per capita/per day; the consumption of the milk is 0.13 L per capita/per day (0.46–0.13 = 0.33 kg of milk products divided by 2 = 0.16 kg of mozzarella or similar cheese (approximately 1 crucible/dose per day).

The contribution to provisional tolerable weekly intake (PTWI) was estimated basing on values available in the literature according to the formula:

$$\mathrm{contribution }\%=\frac{\mathrm{EDI x }7}{\mathrm{PTWI}}x100$$

The contribution to PTWI for Pb and also for Ba, Ni, Sb, Sr, Ni, and Zn was calculated as these elements are grouped into essential elements, but only at very low doses.

### Statistical Analysis

The analysis of variance (one-way ANOVA) was used to determine significant differences in concentrations between groups studied. Statistical software SAS Release 9.1 (SAS Institute Inc. Cara, USA, 2002–2003) was used for all the calculations. The relationships among elements were examined by Pearson’s correlation coefficients. Significance level was set at 0.05.

## Results and Discussion

The concentrations of particular elements and heavy metals in mozzarellas available on the Slovak market are reported in Table 2.

**Table 2:** Mean concentrations (mg/kg) of trace elements in mozzarella followed by SEM

Element	Mozzarella			Whey		
	Classic	Light	Basil	Classic	Light	Basil
Al	0.14 ± 0.04^*a^	0.10 ± 0.03^*a^	0.15 ± 0.03^*b^	0.024 ± 0.004	0.024 ± 0.005	0.010 ± 0.003
As	Below LoD	Below LoD	Below LoD	Below LoD	Below LoD	Below LoD
Ba	1.77 ± 0.29^*a^	1.65 ± 0.24^*a^	2.58 ± 1.32^*b^	0.05 ± 0.01	0.09 ± 0.03	0.01 ± 0.00
Ca	9421.10 ± 1168.38^*a^	9346.98 ± 407.85^*a^	7316.72 ± 219.63^*b^	255.84 ± 61.94	299.77 ± 51.35	366.20 ± 83.72
Cr	0.48 ± 0.10^*^	0.46 ± 0.07^*^	0.43 ± 0.07^*^	0.05 ± 0.01^A^	0.04 ± 0.00^A^	0.02 ± 0.00^B^
Cu	4.24 ± 0.90^*^	5.07 ± 0.60^*^	5.17 ± 0.46^*^	1.10 ± 0.10^A^	1.18 ± 0.19^A^	0.48 ± 0.13^B^
Fe	5.34 ± 1.09^*^	4.69 ± 0.56^*^	6.44 ± 0.77^*^	1.43 ± 0.19^A^	1.34 ± 0.23^A^	0.51 ± 0.12^B^
K	983.11 ± 107.44^*^	991.91 ± 67.17^*^	750.08^*^	204.87 ± 50.13	208.68 ± 10.10	251.87 ± 73.33
Mg	378.01 ± 18.01^*a^	361.37 ± 23.48^*a^	282.42 ± 13.38^*b^	24.11 ± 6.75	26.67 ± 2.59	32.15 ± 8.37
Mn	1.48 ± 0.38^*^	1.33 ± 0.25^*^	1.43 ± 0.29^*^	0.14 ± 0.03	0.44 ± 0.17	0.05 ± 0.02
Mo	Below LoD	Below LoD	Below LoD	Below LoD	Below LoD	Below LoD
Na	7297.78 ± 1075.48^*a^	6163.84 ± 637.78^*^	3721.05 ± 583.06^b^	875.83 ± 317.33	880.18 ± 42.05	1427.43 ± 439.33
Ni	1.49 ± 0.51^*^	1.61 ± 0.35^*^	0.72 ± 0.23	0.19 ± 0.05	0.20 ± 0.13	0.05 ± 0.01
Pb	0.58 ± 0.12	0.81 ± 0.11	0.58 ± 0.33	Below LoD	Below LoD	Below LoD
Sb	1.09 ± 0.16^*^	1.11 ± 0.16^*^	1.38 ± 0.10^*^	0.21 ± 0.01	0.33 ± 0.10	0.10 ± 0.04
Se	0.50 ± 0.14	0.14 ± 0.02	0.37 ± 0.30	0.04 ± 0.03	0.08 ± 0.05	0.01 ± 0.01
Sr	3.69 ± 0.38^*^	3.88 ± 0.33^*^	3.37 ± 0.23^*^	0.03 ± 0.01	0.09 ± 0.03	0.07 ± 0.02
Zn	63.98 ± 2.14^*^	61.42 ± 1.93^*^	66.86 ± 4.69^*^	1.33 ± 0.20^A^	1.42 ± 0.09^A^	0.71 ± 0.12^B^

^*^Means significant differences in row against whey classic, light, and basil, *LoD*, limit of detection. ^a,b^ or ^A,B^Different letters in rows indicate statistically significant differences

Regarding the Al concentration, we found significantly higher level (*P* < 0.05) in basil mozzarella compared to classic and light samples. Significantly lower content was found in all whey samples (*P* < 0.001) in comparison to the solid part of mozzarella. The Al concentration can rise during the steps of cheese manufacturing as Al containers are manufacturing steps that could not be avoided during processing [20]. Probably the addition of basil to the mozzarella required longer processing and contact of food with containers which could have an implication in the increase of Al concentration in mozzarella samples. According to Ranau et al. [21], Al migration depends on some parameters as preparation conditions (temperature and duration of process), chemical composition of food ingredients, pH value of food, and the presence of other compounds and substances (salt, additives, and organic acids).

As is considered a non-essential toxic element [22]. It is used primarily in herbicide and pesticide formulations in agriculture, in numerous industrial processes, e.g., production of semiconductors, pigments, glasses, and textile printing [23]. Concentration of As in milk depends on various factors such as environmental contamination, nutrition, and manufacturing processes [24]. The permissible limit set by the Ministry of Health of the Slovak Republic is 0.25 mg/kg for dairy products and 0.3 mg/kg for hard cheese [25]. In our samples, the content of As was below the limit of detection. In delactosed mozzarella samples from Italy [26], an average value of 0.002 mg/kg was measured for As. Higher level of As in Galician cheese was detected only in cheeses made from cow milk from polluted regions [27].

The Ba concentration in our study showed significantly higher (*P* < 0.05) values in basil solid part of mozzarella in comparison to the classic and light samples. All values in the solid parts (from 1.65 ± 0.24 till 2.58 ± 1.32 mg/kg) were significantly higher (*P* < 0.05) when compared to the whey samples (0.01 ± 0.00–0.09 ± 0.03 mg/kg). Pearson and Ashmore [28] reported concentrations from 0.0001 mg/kg in bottled water to 8.9 mg/kg in bran flake cereal. In UK Total Diet Study, Rose et al. [29] found the concentration of Ba in milk of 0.07 mg/kg, but up to 131 mg/kg in nuts. WHO guidelines [30] establish a TDI for Ba in drinking water in the amount 20 µg/kg. However, in epidemiological studies, water containing 7.3 mg/kg Ba was considered to have no adverse influence on the consuming population. On the contrary, WHO assessment did not determine the dose at which adverse or risk effect may be expected.

Among essential mineral elements, Ca is the most common in milk [10]. The Ca contents in classic and light mozzarella samples were significantly higher (*P* < 0.05) when compared to basil. Significantly lower values (*P* < 0.05) were found in whey groups in comparison to the solid parts. In our previous study with cottage cheese [16] and milk [14], we noticed lower values of Ca concentrations (1152.00 ± 33.04–1375.62 ± 90.62 mg/L and 3196.82 ± 1828.25–3789.73 ± 1095.53 mg/L). Highest quantities of Ca were found in hard cheeses (Gouda, Edam, and Parmigiano) and the lowest in cream and cottage cheese [10]. Samara et al. [7] observed favorable impact of milk, yogurt, and cottage cheese consumption on metabolic profile and anthropometric indices in normal-weight men. Ca and Mg are the most important elements for building teeth and bones and to avoid osteoporosis in human population [31]. Ca is responsible for many regulatory functions, such as blood clotting, secretion of hormones, cardiac rhythm, muscle contraction, and enzyme activation [9]. The intake of dairy products is more efficient in regards to Ca content than Ca supplementation due to the presence of lactose that improves Ca assimilation [32, 33]. The majority of dietary Ca comes from milk and milk products (about 70%) because in milk, casein micelles contain the natural vector of Ca [34].

In our samples, the concentration of Cr in the solid part was significantly (*P* < 0.05) higher when compared to liquid part. In liquid parts of basil whey, the concentration of Cr was significantly lower (*P* < 0.05) in comparison to the classic and light whey. Cr was detected at high level in sheep milk in Southern Italy [35].

The concentration of Cu did not show significant differences (*P* > 0.05) in the solid parts of the mozzarella, but significant differences were found in the whey samples. Significantly lowest (*P* < 0.05) amount of Cu was found in basil whey compared to classic and light samples, and at the same time, Cu concentrations were significantly lower (*P* < 0.05) in all whey samples compared to the solid parts of the cheese. Cu is an essential element required for normal physiological functions in the human body [20]. However, in our case, all examined solid parts of mozzarella exceeded the permissible limit (0.25 mg/kg for dairy products) set by the Ministry of Health of the Slovak Republic [24] and ranged from 4.24 ± 0.90 to 5.17 ± 0.46 mg/kg. Since the consumption of mozzarella is not so widespread in the Slovak Republic, the higher amounts of Cu in our samples do not pose a threat to human health. Similar results were obtained by Deeb [36] in feta cheese (4.14 ± 0.25 mg/kg) and by Elbarbary and Hamouda [20] in fresh feta cheese (3.25 ± 1.06 mg/kg). The increased Cu intake may be a hazard to human [37]. Toxic level of Cu and its long term consumption can lead to Wilson disease characterized by excessive accumulation of Cu in liver, brain, kidney, and cornea [38]. On the contrary, in our previous study with yogurt [15], milk [14], and cottage cheese [16] from the Slovak market, the level of Cu was found under permissible limit. Level of Cu increased from 0.45 mg/kg in raw sheep milk to 0.78 mg/kg after curdling of milk because of its ability to bind with casein [39]. Lante et al. [40] recorded significantly higher values of Cu in butter than in milk, kareish cheese, and rice pudding as the result of the fact that it is preferentially bound to proteins and membrane lipoproteins of milk fat globules.

Fe belongs to the essential elements and partakes as catalyst in some metabolic reactions. It plays an irreplaceable role in the transport, storage, and utilization of oxygen as a component of hemoglobin, myoglobin, cytochromes, and other proteins. Its deficiency can result in anemia [13]. One of the most common nutritional deficiencies in childhood is Fe deficiency because of quick growth. However, cow milk and dairy products are considered poor sources of Fe because the bioavailability of Fe from cow milk ranged from 10 to 34% [9]. Significantly lower level (*P* < 0.05) of Fe was found in each whey in comparison to the solid parts. We found significantly lower values (*P* < 0.05) in the basil whey when compared to the classic and light whey. The Fe contents in the solid mozzarella parts were higher (ranged from 4.69 ± 0.56 till 6.44 ± 0.77 mg/kg) than those published in our previous results in milk (1.76 ± 0.64–1.92 ± 1.45) and cottage cheese (1.16 ± 0.14–2.67 ± 0.59) [14–16]. Higher level of Fe (7.47 mg/kg) was reported in kareish cheese [36].

Mg has an important role in various physiological processes, such as metabolism of proteins and nucleic acids, muscle contraction, neuromuscular transmission, blood pressure regulation, and bone growth. It is also co-factor of some enzymes, and its deficiency may also lead to osteoporosis [41]. In our study, the concentration of Mg was significantly lower (*P* < 0.05) in basil mozzarella in comparison to classic and light samples. In whey samples, values of Mg were significantly lower (*P* < 0.05) as those measured in the solid parts. In our previous study, the content of Mg in milk [14] and cottage cheese [16] was lower (from 202.70 ± 10.83 to 273.23 ± 16.32 mg/l) than in mozzarella samples (from 282.42 ± 13.38 to 378.01 ± 18.01 mg/kg).

The Mn content in our samples ranged from 1.33 ± 0.25 to 1.48 ± 0.38 mg/kg in the solid parts and significantly lower values (*P* < 0.05) from 0.05 ± 0.02 to 0.44 ± 0.17 mg/kg were detected in whey samples. Al Sidawi et al. [42] conducted a monitoring study in Georgia and measured in Imeruli cheese the average value of Mn 0.86 ± 0.595 mg/kg and in Salguni cheese 2.348 ± 2.267 mg/kg.

It is known that particularly high level of Na is detected in cow colostrum. The Na content in the milk declines to an average level a few days later and is higher at the end of lactation period [9]. In our samples of mozzarella, the classic sample had the highest Na content, while the basil sample had the lowest Na content. The difference between classic and basil solid parts of mozzarella was significant (*P* < 0.05). Significantly lower values (*P* < 0.05) were measured in all whey samples when compared to the solid parts. Higher values (about 8083 mg/kg) were recorded in traditional Slovak cheese named Oštiepok [43]. The concentration of Na is related to K concentration in milk of cows, especially during the lactation period. Hypokalemia can be present in critically ill cattle in respect to clinical disorders such as hepatic lipidosis, abomasal displacement, volvulus, or downer cow syndrome [44]. The depletion of K stores in the organism is evident during marked metabolic acidosis in cows [45]. In our samples, we found significantly lower values (*P* < 0.05) of K in all whey samples in comparison to the solid parts of mozzarella.

The Ni content showed significantly higher (*P* < 0.05) amounts in classic and light mozzarella samples in comparison to all whey parts. Similar results were obtained in our previous study with milk [14]. The permissible limit given by the Ministry of Health of the Slovak Republic is 0.1 mg/kg for milk and milk products [25]. In our case, the values of Ni were above the limit in mozzarella samples (0.19–1.61 mg/kg). In our other study on cottage cheese, we found lower values (0.09–0.27 mg/L) of Ni in samples available at the Slovak market [16]. Higher values in mozzarella are probably the results of industrial processing of the milk. But, seeing that the consumption of mozzarella in the Slovak republic is not so frequent, the higher amounts of Ni in our samples do not pose any threat for human health. Al Sidawi et al. [42] obtained the concentration of the essential trace elements in the cow milk samples from the highest value in the order Cr, Mn, Ni, Se, and Mo and in the cheese samples Se, Mn, Mo, Ni, and Cr. In our samples, we observed the order of Ni, Mn, Se, Cr, and Mo. In the case of Mo and As, we determined values below LoD in all mozzarella samples. In the Total Diet Study in New Zealand [28], Ni was widespread and prevalent mainly in plant-based foods. The European Food Safety Authority [46] assigned non-alcoholic beverages as an important dietary source of Ni. For risk assessment of Ni, EFSA established the TDI for Ni of 2.8 µg/kg bw/day.

The Pb concentration in the solid parts of mozzarella ranged between 0.58 ± 0.12 and 0.81 ± 0.11 mg/kg in contrast to whey where the values were below the detection limit. According to Elbarbary and Hamouda [20], Pb was the highest element in fresh cheese which showed a significant increase after curdling. Similar tendency was obtained in sheep and goat milk [39]. The permissible limit given by the Ministry of Health of the Slovak Republic is 0.5–0.7 mg/kg [25]. In our case, the values of Pb were slightly above the limit. Lower values (0.01 ± 0.002–0.07 ± 0.002 mg/L) were noticed in our previous results in cottage cheese [16] and values in interval 0.11 ± 0.04–0.36 ± 0.06 mg/kg in yogurt [15]. Cheese can be contaminated with Pb from the contaminated salt, lead piping, and lead-linked tanks [20], also by environmental conditions at the processing sites [35]. Sizova et al. [47] found that indoor and outdoor rations have an important impact on trace element and mineral metabolism in dairy cows after their occurrence in milk. Aggregated forms of chemical substances (e.g., heavy metals) typify a severe risk in foodstuffs because of its long term toxicological effects. However, when the intake of essential elements exceeds the recommended quantities markedly, they can have harmful effect and may become toxic [20].

The Se content in the solid parts of mozzarella ranged from 0.14 ± 0.02 to 0.50 ± 0.14 mg/kg and in whey from 0.01 ± 0.01 to 0.08 ± 0.05 mg/kg without significant differences (*P* > 0.05) among the groups. Se content in milk can be affected by geographic factors, stage of lactation, and nutrition [48]. In the study raw milk and mozzarella cheese [1], the content of Se was higher in cheese than that in milk. The Se in cheese should come from the fat and casein as most of the whey is drained during the cheesemaking. It is known that Se is an important trace element. Its deficiency is associated with negative effects in animal and human health [1].

Sb is a nonessential toxic element, but some data presume its biological essentiality [24]. Sb is exploited in industry, e.g., in glass, paints, rubbers, fireproof textiles, and in alloys with Pb, Cu, and stannum (Sn) [49]. Food legislation on the presence of metallic elements in foods of the Slovak Republic [25] establishes a maximum tolerated level for Sb from 0.05 mg/kg in milk products to 0.3 mg/kg in other food products. In our samples, this limit was excessed in dry matter (1.09–1.38 mg/kg). Significantly lower (*P* < 0.05) values were found in all whey samples (0.10–0.33 mg/kg). However, due to the limited frequency of consumption and the amount used in food preparations, it is important to note that consuming mozzarella in moderation does not pose a threat to the consumers. Belzile et al. [50] indicate that a possible source of Sb in milk products could be the use of milk containers or tanks treated with paint, enamel, ceramic, or plastic surface. The hazard characterization of Sb revealed a TDI of 6 µg/kg bw/day in drinking-water [51].

The Sr contents in our study were significantly (*P* < 0.05) higher in the solid part of mozzarella than those found in the whey parts. The concentrations of this element were lower than those reported by Sanal et al. [52], 6.44 mg/kg and 6.13 in milk of sheep and goats. Sr is a Ca agonist; it adheres to the bone surface and it is involved in the formation of the mineral portion of the bone and stored in the body for many years. The lack of Ca can be a critical issue for children and individuals during their growth period. If children diet is deficient in Ca and protein, it can result in poor bone growth [53]. For Sr, a TDI of 130 µg/kg bw/day was set [54].

The Zn concentration in mozzarella ranged from 6.14 ± 0.19 to 6.69 ± 0.47 mg/kg without significant differences (*P* > 0.05) among the groups. Lower values were obtained in our previous study [15, 16] with cottage cheese (from 1.80 ± 0.08 to 2.20 ± 0.15 mg/kg) and yogurt (from 0.91 ± 0.13 to 3.64 ± 0.10 mg/kg). Meshref et al. [13] reported similar mean concentration of Zn in cow milk 6.29 mg/kg and higher in kareish cheese 8.59 ppm. It is known that only 1–3% of Zn in milk is associated to the lipid fraction and 97% is related to casein fraction and can be found in the skim milk fraction and subsequently shift to the curd during dairying [40]. In our study, significantly lower values of Zn (*P* < 0.05) were found in whey samples (0.07 ± 0.01 to 0.14 ± 0.01 mg/kg) in comparison to the solid parts of mozzarella. From the whey samples, the lowest value (*P* < 0.05) was found in basil whey against classic and lights samples. All examined parts of mozzarella were within the permissible limit (50 mg/kg for cheese and 25 mg/kg for milk products) given by the Ministry of Health of the Slovak Republic [25]. Zn is essential mineral requisite for the structure and activity of many enzymes responsible for nucleic acid and protein synthesis, cellular replication and differentiation, sexual maturation, insulin secretion, antioxidant defense, and other physiological processes [8, 55]. In general, animal food products have a higher Zn content compared to vegetables [55].

### Risk Assessment of Consumers

An uneven spreading of elements between whey and the solid part of cheese depends on the different element binding forms in the milk. It has been observed that curdling allows the increase of concentrations of some elements (e.g., Cu, Pb, and Cu) in the curds in comparison to raw milk. These elements are preferentially bound to casein and fat and then shift mostly to the curds [20, 39]. Aggregated or inorganic forms of chemical substances as heavy metals or metalloids denote a severe risk in foodstuff for its long term toxicological activity. Moreover, when the consumption of essential elements exceeds the recommended amounts considerably, they will have a detrimental effect and become toxic [38].

The provisional tolerable weekly intake (PRWI) for particular elements is officially established by the European Food Safety Agency [56]. It means the acceptable concentration of some toxic or biologically active elements that can be consumed on a weekly basis. It was settled for the purpose of estimating the potential risk to human health resulting from the food and accumulation of particular toxic elements on a weekly basis [57]. Our results showed that Ba, Ni, Sb, Sr, Pb, and Zn are in higher concentration in the solid part of mozzarella compared to whey samples. Contribution to PTWI depends on the amount of food consumed and the level of contamination [57]. In our study, the highest contribution to PTWI was calculated for Ni in the solid parts of mozzarella (29.8%, 32.2%, and 14.4%). According to the fact that Ni is essential element and it is toxic only in high amount, we can conclude that PTWI for Ni in mozzarella samples did not show a dangerous level for human health, also due to the fact that mozzarella is not consumed in large quantities in Slovakia. Pb as a toxic element showed very low concentrations in the mozzarella samples and did not contribute significantly to the PTWI. In milk from Serbia, Pb accounted for a higher percentage (1.37%) of contribution to PTWI [54]. According to Manzi et al. [55], the consumption of traditional Italian cheeses did not pose a potential risk to public health, whereas none of the cheeses examined exceeded the provisional maximum tolerable daily intake for Zn. The comparison of average values of particular elements, EDI and PTWI, suggested that consumption of mozzarella in the Slovak republic does not pose a threat to human nutrition (Tables 3 and 4). The consumption of this kind of cheese can contribute to the daily intake of important nutrients as Ca, Mg, and others. Similar results were achieved in our previous study with milk [14] and cottage cheese [16]. Likewise, in study of Crupi et al. [26] regarding the consumption of delactosed mozzarella, any safety risk due to exposures to toxic elements was excluded. Galician cheeses were found to have a low level of toxic metal residues [27]. Generally, mineral elements ingested through the diet play an important role in the human body, and most biochemical reactions depend on or are influenced by these electrolytes. Anthropogenic activities can significantly increase the content of trace elements in the environment and consequently enter the food chain. A healthy diet containing sufficient amounts of various electrolytes and essential elements can have great potential to prevent or alleviate many health problems. Much attention has been given for identifying the recommended daily intake because a number of health risks have been associated with either deficiency or excessive intake [28, 58–60].

**Table 3:** Estimated daily intake (EDI) of elements studied in the mozzarella available on Slovak market (calculated for 70 kg person)

Element	Classic	Light	Basil	Classic whey	Light whey	Basil whey
Ba	1.77	1.65	2.58	0.05	0.09	0.01
Ni	1.49	1.61	0.72	0.19	0.2	0.05
Sb	1.09	1.11	1.38	0.21	0.33	0.1
Sr	3.69	3.88	3.37	0.03	0.09	0.07
Pb	0.58 × 10^−7^	0.81 × 10^−7^	0.58 × 10^−7^	0.00 × 10^−7^	0.00 × 10^−7^	0.00 × 10^−7^
Zn	63.98	61.42	66.86	1.33	1.42	0.71

EDI values calculated on the basis of 160 g consumption per person (70 kg) per day

**Table 4:** Contribution to provisional tolerable weekly intake (PTWI) of elements studied in the mozzarella available on Slovak market (calculated for 70 kg person)

Element PTWI (µg/kg bw/week)		Contribution to PTWI (%)					
		Classic	Light	Basil	Classic whey	Light whey	Basil whey
Ba	140 ^(WHO, 2004)^	8.85	8.3	12.9	0.25	0.5	0.1
Ni	35 ^(EFSA, 2010)^	29.8	32.2	14.4	3.8	4.0	1.0
Sb	42 ^(WHO, 2003)^	18.2	18.5	23.0	3.5	5.5	1.7
Sr	910 ^(WHO, 2010)^	2.8	3.0	2.6	0.02	0.1	0.1
Pb	25 ^(Shahbaziet al., 2016)^	0.0	0.0	0.0	0.0	0.0	0.0
Zn	7000 ^(EFSA, 2010)^	6.4	6.1	6.7	0.1	0.1	0.1

## Conclusion

Our work presents a comprehensive study of the content of essential and non-essential elements in the solid and whey parts of mozzarella available on the Slovak market. The consumption of mozzarella in the Slovak republic is slowly increasing due to the positive attitude and perception of Italian cuisine, and its consumption can contribute to the intake of important essential elements such as Ca and Mg, along with others. The contents of some toxic and trace elements were low and did not exceed the permitted limit. In addition, the contribution of Ba, Ni, Sb, Sr, Pb, and Zn to PTWI was found to be very low, indicating that the consumption of mozzarella does not pose a higher risk to humans. The data generated through this study can serve as a valuable addition to existing methodological and research material in the field of dairy food safety and can contribute to a better understanding of the positive impact of food safety practices on human health.

## Data Availability

Not applicable.
